# Supplementary material for: Development of a Set of Wheat-Rye Derivative Lines from Hexaploid Triticale with Complex Chromosomal Rearrangements to Improve Disease Resistance, Agronomic and Quality Traits of Wheat
Source: Plants (Basel). 2023 Nov 17;12(22):3885. doi: 10.3390/plants12223885 (PMC10674216; doi:10.3390/plants12223885)
Supplement: Supplementary file 1 [file plants-12-03885-s001.zip › plants-2683813-supplementary.pdf]

Supplementary Materials

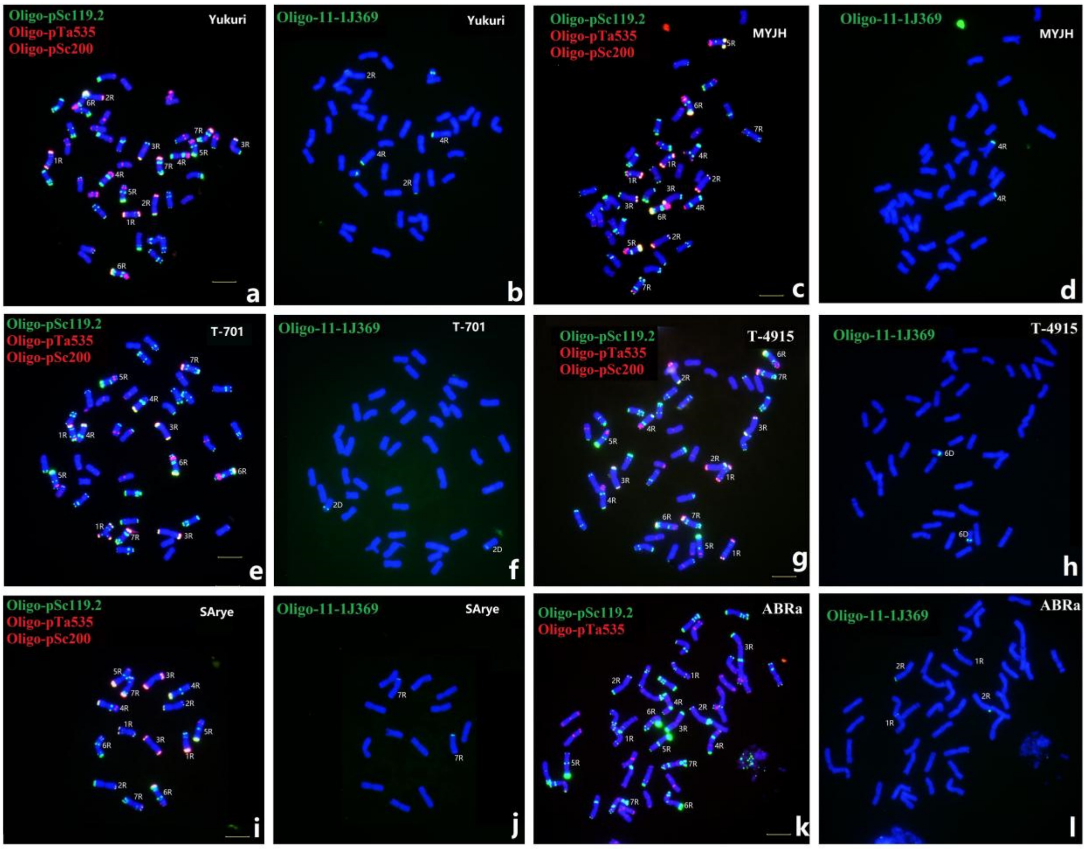

Figure S1. Comparative ND-FISH between Yukuri and other triticale and rye lines.The probes of Oligo-pSc119.2 + Oligo-pTa535 + Oligo-pSc200 (a, c, e, g, i, k), and sequential probe of and Oligo-11-1J369 (b, d, f, h, j, l) are shown. The lines Yukuri (a, b), MYJH (c, d), T-701(e,f), T4915 (g, h), SArye (i, j), and durum wheat-S. africanum amphiploid YF (k, l) are indicated. Bar 10μm.

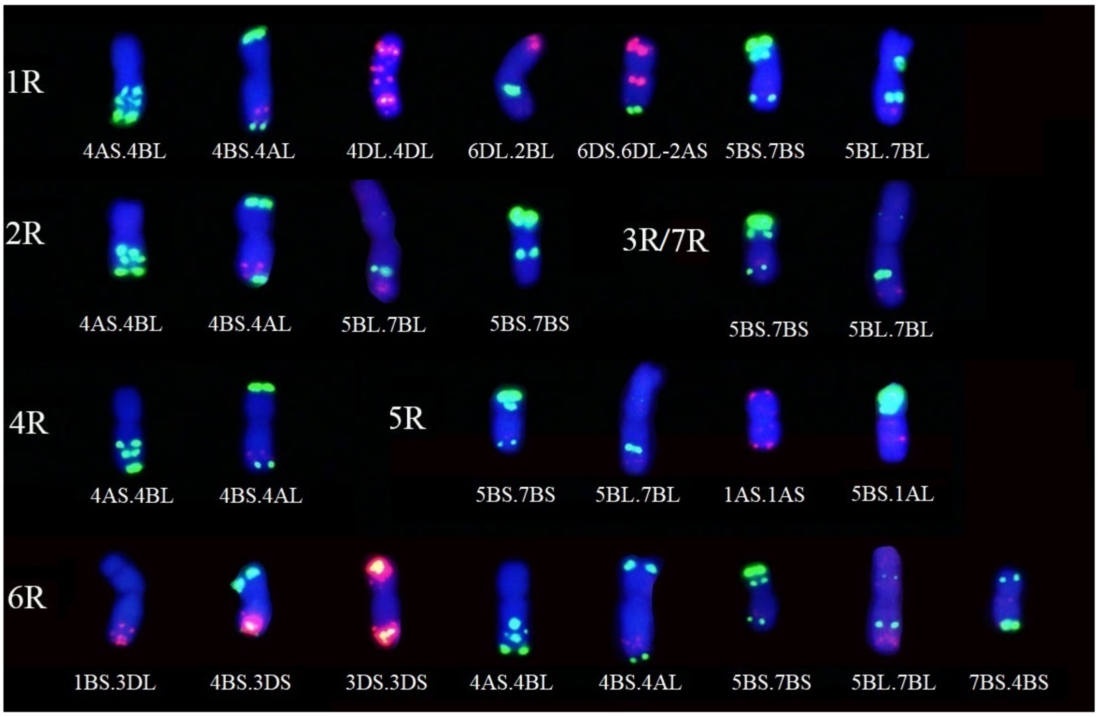

Figure S2. Different types of modified wheat chromosomes in wheat-rye additions. The ND-FISH patterns of Oligo-pTa535 (red) and Oligo-pSc119.2 (green) are indicated.

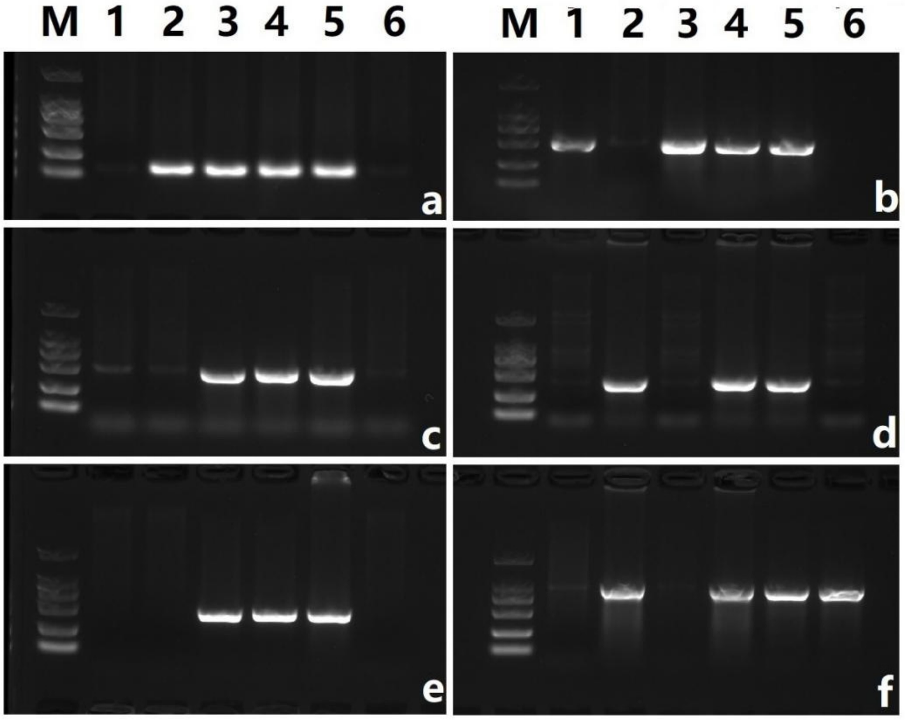

Figure S3. The amplification of chromosome 1R specific PCR based molecular markers. (a-c) 1RL specific markers 1RL673 (a), Ku1RL248 (b) and Ku1RL589 (c), (d-f) 1RS specific markers Ku1RS127 (d), 1RS15.3 (e) and 1RS2.29 (f) were used for PCR. The plant materials 1-6 were CS, JH, CSDA1R, Yukuri, MA1R (wheat-monosomic 1R addition from Yukuri), AK58, respectively. M, markers.

Table S1. The sequences and hybridization sites of the novel probes by ND-FISH

Name of probe	Sequences (5'-3')	Hybridization sites on wheat chromosome	Hybridization sites on rye chromosomes
Oligo-pSc119.2	CCGTTTTGTGGACTATTACTCACCGCTTT GGGGTCCCATAGCTAT	1A-7D	1R-7R
Oligo-pTa535	GACGAGAACTCATCTGTTACATGGGCAC TTCAATGTTTTTTAAACTTATTTGAACTC CA	1A-7D	
Oligo-pSc200	CTCACTTGCTTTGAGAGTCTCGATCAATT CGGA CTCTAGGTTGATTTTTG TATTTTCT		1R-7R
Oligo-Ku	GATCGAGACTTCTAGCAATAGGCAAAAA TAGTA ATGGTATCCGGGTTCG		1R-7R
Oligo-K288	CTTCATAGTCCGGGAGTCCGGCCAAAGG TCATAGTCCG GCCATCC	1A-7B	

Oligo-5SrDNA	TCAGAACTCCGAAGTTAAGCGTGCTTGGGC GAGAGTAGTAC	1BS, 1DS, 5AS, 5BS, 5DS,	5RS
Oligo-3A1	AATAATTTTACACTAGAGTTGAACTAGCTCTATA AGCTAGTTCA	3AL，5DS，5AL，5BL，7AS，7AL，	5RL
Oligo-V03-71	CTTCTCCGAGTGAAGAGCGATCCTCTCACTCGGGGGCTTAGCTGCGAGTCTAATC	4BL，7BS，5A，6DS，7AS，6BL，5BS，1BS，1AS	1R，4RS，4RL，7RL
Oligo-StC12	ATGTTCTATCACCCATCCCGCTACCACCCTCACTTTTTCACCAG		1RS，4RS，5RS，6RS，7RL
Oligo-13-J1011	CATCATGCTTGTTGTGAGAAGCTCTGGTTTGTGAGAAGCATATACCCAAACC	6D，7A，1A，2A，4A	1RS，3RS，6RS，7RS
Oligo-11-1J369	GAGTTCGTTCAGAAACTTGTATAGAAACTTTCTTAATGAGCACTCGACA	6DS	2RS，4RS
Oligo-09-4St142	GGATCGCTCTACACTCGGAGAAGTTTTTAACTTAGACGAAAGCTGGTTCGTA	6BL，1B，7B	2RS，5RS
Oligo-6E-571	ACGTAACGATTTTAGAGGCTAGGAGTGGACACGCCAGCGATTAAATCATC		1RS，6RS，7RS
Oligo-2S-119	TTGTTTTCGCCGTGTGGCATGCTGTAACTTATGTGTGTGACAGCCAAGAACCAAAGGGC		2RS，4RS
Oligo-6E-376	CGTATTTCAAATCATACGGACCTAAAAATTAGAAGTCATTCCATCTTTA		1R，2R，3RL，4RL，5R，6R，7R
Oligo-P05	AATACGCTCTTGTTCTTGGCTGTCACGCACATACTTTATGGGATGTCATAGG		2RS，4RS
Oligo-2S-248	AGGAGTTCTTGGGCGACCTTTGTTTCAGAGTGACGTGTGGTAGGGTTTAGGGCAAAT	6BS，3BL	4RL，6RS
Oligo-7E-369	AACTTAGGCGAGGACTGGCTCGCGGCTAAGCCCCCGAGTGAGAGGGATGCTCTCA	6BL，4BL，7BS，5A，6DS，7AS，5BS，1BS，1AS	4RS，4RL，7RL
Oligo-St96-1	GAGCTGCTATGCACATTTTCGTCCAAGAACGGCGCATGTTCTT		1RS，4RS，5RS，6RS，7RL
Oligo-St96-2	CAGGAACTCAATTTTGCAAGCCGGTTTTGGCCTCCGAAACATT	6B，3B，1BL，4BL，5BS，7B	1RS，2RS，4R，5R，6R，7R
Oligo-2S-163	GAGCACACGTGCGGTAAGTCTCGGCCCCAGAAAAGGCCGTTTAGACCCTCAAAAAGGCT		1R，2R，3RL，4RL，5R，6R，7R
Oligo-D01-135	ACGCGCGCCATGGAAAACAGGGCAAAACCACCGACTCGTCCACGACTCGTAC	1BS，6BS	1RS
Oligo-StC11	TTGAGTTTCGGATGATTTTATCTTTGGCGTGTACTCTGCCACCCT	6BL	1RS，4RS，5RS，6RS，7RL
Oligo-7E-553	CGGGGGCTTAGCCGCGAGCCAGTCCTCGCCTAAGTTAAAAAATTAC	4BL，7BS，5A，6DS，7AS，6BL，	1R，4RS，4RL，7RL

		5BS，1BS，1AS	
Oligo-TB553-2	TTTGTAATGGAAGGATGGCGCATTGTTC TATATGTTATTGTCCATATA		1RS，6RS，7RS
Oligo-10-7J89	CAGCCTGAAGTGAAGAGATCTCCCGCTG AGCTTCCGCAGCGGCC		1R，2R，3RL，4RL，5R，6R，7R
Oligo-7E-716	GTACAGGACTGCAGCTAAGCCCCCGAG TGAGAGGGTTGCTCATCACTCGGTAGGA TT	6BL，1B，7B	2RS，5RS
Oligo-Y372	AATTTCGTCACCGTATCTTCTCTAATGTG ACACCAACACCCTATCGTAGACATCCGT ATCT		1R，2R，3RL，4RL，5R，6R，7R
Oligo-$(GAA)_7$	GAAGAAGAAGAAGAAGAAGAA	1B-7B，2A-7A，2D，7D	2RL，3RS，6R
Oligo-$(CAA)_7$	GAAGAAGAAGAAGAAGAAGAA	7A， 3D， 2A，1B-7B，4A，4D	1R-7R
Oligo-1B-3	CAATGGGTTCAGAAACTTGTCTGAAAAA CAGCGACGAATGATG		2RS，4RS
Oligo-2H-18-50	TAATAAGCTTAGTTAGCTCCAAAATGAC ATATTTTC	6BL，7BL，3BS，	5RS，7RL
Oligo-pTa1-360	CCTTCAAAAGGAAGTGCCAATGGGTTCA GAAACTTGTCCGAAAAACAGCGGCGAA TG		2RS，4RS
Oligo-pAWRC	CGTAGGCGCCGATCTTGAAAGAGACTTG CACGGTGTGCTCGACTCGAAGAATTCCG GCGT		1R-7R
Oligo-CCS1	CCGTTTGATAGAGGCAAAGGTGTCCCGT CT TTTGATGAGA	1A-7D	1R-7R
Oligo-pTa1-335	TCTATAATCAACATAATCTCTCGAATTT CCTTCAGAAGGAAGTGCCAATGGGCTC AG	6BL，1B，5B	2RS，4RS

Table S2. Sequences and location of the 1R-specific markers

Primers	Forward	Reverse	Location on 1R of Lo7 (Mb)	Reference
1RS2.29	GTTGCTCCCATGGCAT GACAG	GCTGGTAGTTAATG CACCGAG	2.29	This study
1RS15.3	GTTTGGAGCGATAAC TAGTGA	GGAACTGGTGGATA CGGTTC	15.30	This study
KU1RS127	TGAGATGAGAGAGGG GTCCT	AATGTGGAGGGTAG CTGCAA	139.89	Qiu et al. (2016)
KU1RL589	GCACAAGATGTAGCC	GCACGTAATTTGGC	535.09	Qiu et al.

	AGCTG	GATGG		(2016)
1RL673	GGTGCGTCTTAAACT GATTC	GATCGTAGTGGGTA GTATCT	673.99	This study
KU1RL248	TTTCAGAGAGGCATG AATGCTA	CCCTCAAAGGCGAT TTTACA	685.49	Qiu et al. (2016)
